# Weight loss is linearly associated with a reduction of the insulin response to an oral glucose test in Icelandic horses

**DOI:** 10.1186/s12917-020-02356-w

**Published:** 2020-05-24

**Authors:** Julien Delarocque, Florian Frers, Korinna Huber, Karsten Feige, Tobias Warnken

**Affiliations:** 1 Clinic for Horses, University of Veterinary Medicine Hannover, Foundation, Bünteweg 9, 30559 Hanover, Germany; 2 Institute of Animal Science, Faculty of Agricultural Sciences, University of Hohenheim, Fruwirthstraße 35, 70593 Stuttgart, Germany

**Keywords:** Insulin dysregulation, Equine metabolic syndrome, Weight loss, Obesity, Oral glucose test, Laminitis, Horse

## Abstract

**Background:**

Insulin dysregulation (ID) goes along with lasting or transient hyperinsulinemia able to trigger equine laminitis, a painful and crippling foot condition. Promoting weight loss through dietary changes and physical activity is currently the main option to prevent this disease. This study aimed at describing the relationship between weight variations and the level of ID as determined by oral glucose tests (OGT). Therefore, the insulin response of 19 Icelandic horses to repeated OGTs was retrospectively analysed considering the variations in their body weight.

**Results:**

There was a strong linear relationship between variations in body weight and variations in the total insulin response to OGT as approximated by the area under the curve of insulin (*p* < 0.001). As indicated by a weighted least squares model, the insulin response decreased by 22% for 5% weight loss on average. However some horses did not respond to weight loss with a reduction of their insulin response to OGT. Additionally, a high correlation between 120 min serum insulin concentration and total insulin response was observed (*r* = 0.96, *p* < 0.001).

**Conclusions:**

The results corroborate that weight loss is effective against ID and allow for a better quantification of the expected improvement of the insulin response after weight loss. However, it is unclear why some horses did not respond as expected. The high correlation between the 120 min insulin concentration and total insulin response suggests that insulin status can be accurately determined and monitored with only few samples in a practical setting.

## Background

Since obesity was identified as a risk factor for laminitis [1, 2], the metabolic health of obese horses has been studied intensively. Eventually, the Equine Metabolic Syndrome (EMS) was defined as a collection of risk factors for this disease, among which insulin dysregulation (ID) plays a major role [3–5]. The term ID encompasses insulin resistance (IR), exaggerated insulin response to oral carbohydrates and fasting hyperinsulinemia (HI) and therefore results in either permanent or transient HI. As HI was shown to induce laminitis [6, 7], it was sought to increase insulin sensitivity (SI) in horses affected by EMS through various weight loss programs [8]. These more or less complex programs relied on increased physical activity [9, 10], restricted energy intake [11–15] or both [15–17]. While combined programs were effective, SI did not necessarily improve when either activity or dietary changes were absent [9, 10, 15]. However, comparing these studies is challenging, because of dissimilar methodologies and a vast number of involved variables (e.g. breed, age, energy source, exact energy intake and expense, etc.). Moreover, it was suggested that obesity is not in itself responsible for a reduced SI in horses [18], which is consistent with the description of a lean EMS phenotype [5, 19] and supports the existence of contributing factors to EMS distinct from IR and obesity.

As HI also occurs in insulin sensitive horses [19, 20], the assessment of SI – a measure associated with IR – is probably insufficient to evaluate the effects of weight loss on ID. While the term ID had not been introduced at that time, the effect of weight loss on the area under the curve of insulin over time (AUC_ins_) during an oral glucose test (OGT) has already been investigated by Van Weyenberg et al. in 2008 [11]. The AUC_ins_, which can be seen as an approximation of the total quantity of insulin secreted during a period of time [21, 22], is a good descriptor of the level of HI induced by a carbohydrate challenge. Further was the use of an oral testing protocol judicious, since these are sensitive to all aspects of ID, contrary to intravenous tests [5].

Five Oral Glucose Tests (OGT) were conducted in 19 Icelandic horses over 1 year during a longitudinal study. As substantial variations in body weight were noticed, it was retrospectively decided to describe the relationship between weight variations and insulin response within each horse to investigate the effect of weight loss on ID.

## Results

### Weight and AUC_ins_ variations

**Fig. 1:**
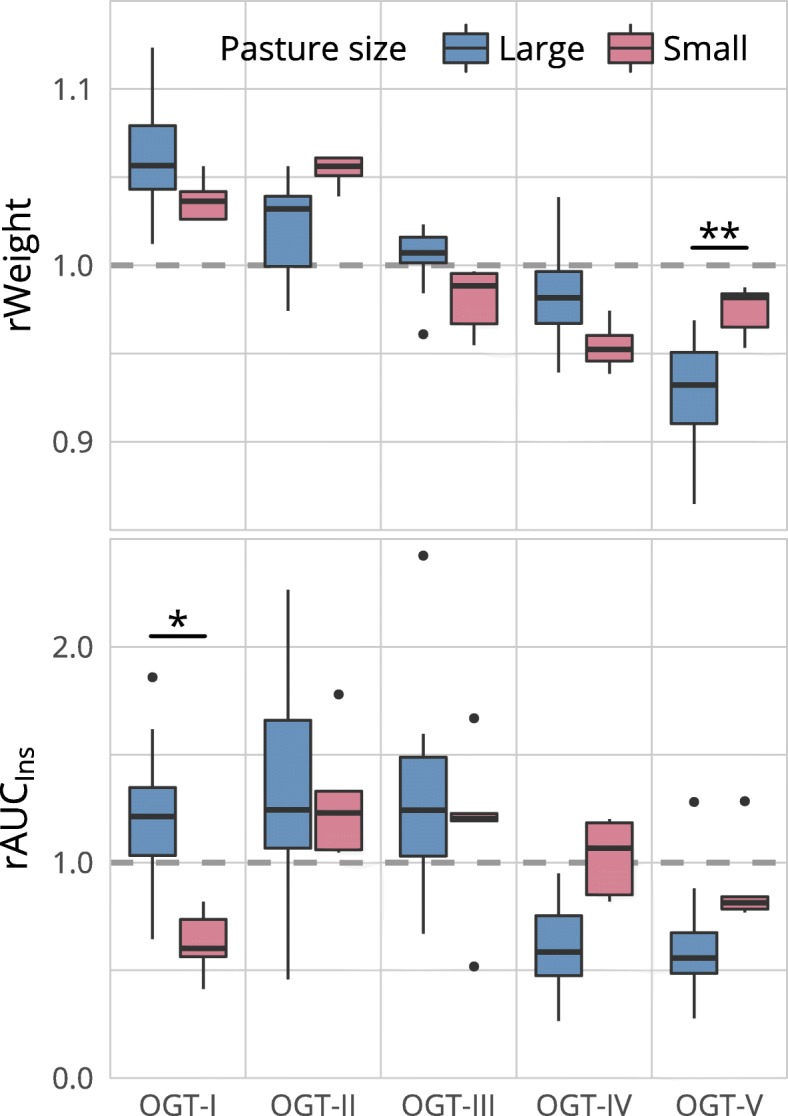
Variation in time of the relative weight (rWeight) and relative area under the insulin curve (rAUC_ins_). The grey dotted line represents the mean weight resp. rAUC_ins_ over the year. The pairwise comparisons between groups for rWeight and rAUC_ins_ reveal that significant differences can only be found at one OGT in each case. However, no significant overall group differences were observed

All horses lost weight over the year (*p* < 0.001). However, the body weight did not monotonically decrease in all individuals. The median maximal weight difference observed within one horse was 12% (45 kg) on the large pasture and 11% (41 kg) on the small pasture. No overall pasture differences were found (*p* > 0.9). The pasture-time interaction (*p* = 0.004) resulted in a significant difference at only one OGT (Fig. 1).

Variations within time were also present for rAUC_ins_ (*p* = 0.004) but no effect of pasture was observed either (*p* > 0.9). The pasture-time interaction (*p* = 0.03) was associated with a significant difference at a single OGT (*p* = 0.01) (Fig. 1).

### Relationship between relative weight and relative area under the curve of insulin over time

**Fig. 2:**
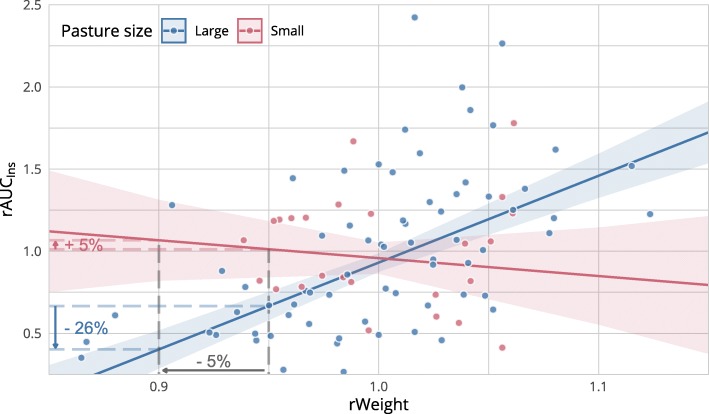
Scatterplot representing the relationship between the relative weight (rWeight) and the relative AUC_ins_ (rAUC_ins_). The regression lines with 95% CI from the weighted least squares model are presented in blue for the larger pasture and in red for the smaller one. On the large pasture a reduction of the relative weight of 5% led to a reduction of the rAUC_ins_ of 26%, while in the other group the insulin response was rather stationary despite weight loss

**Fig. 3:**
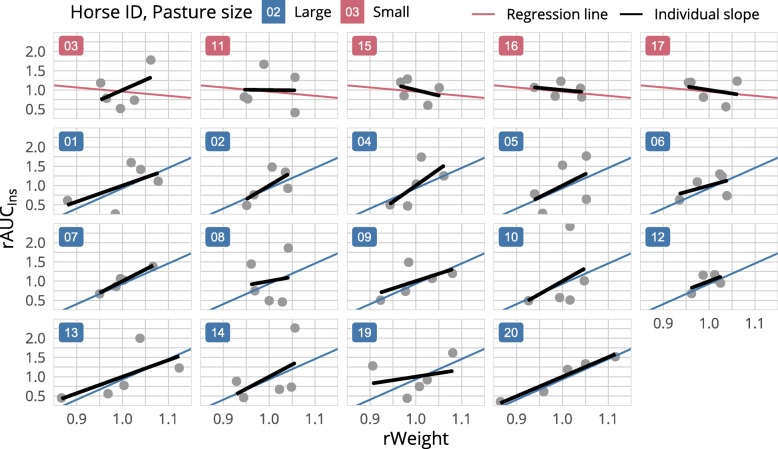
Scatterplot of the relative weight (rWeight) and associated relative AUC_ins_ (rAUC_ins_) for every horse. The coloured lines correspond to the regression lines of the full weighted least squares model presented in Fig. 2. The black line is the individual trend as determined by simple linear regression. On the small pasture the average evolution of the insulin response to weight loss is almost stationary, except for horse 3, whose response is rather similar to the one observed on the large pasture. Possible reasons for these differences by pastures are discussed in “Possible causes for the lack of response in some individuals”

In a first model with no distinction of pastures, the relative insulin response, as approximated by rAUC_ins_, was significantly predicted by rWeight (β = 4.4, SE = 0.6, AIC = 91.9, Number of observations = 95, *p* < 0.001). Overall, 5% weight loss were associated with a reduction of the insulin response of 22%. The addition of pasture to the model improved model fit (Χ^2^(2) = 16, *p* < 0.001). The effect of rWeight increased (β = 5.3, SE = 0.6, AIC = 79.8, Number of observations = 95, *p* < 0.001) but was inversed for the small pasture (β = − 6.4, SE = 1.4, *p* < 0.001). The effect of pasture was significant as well (β = 6.4, SE = 1.4, *p* < 0.001). As a result, 5% weight loss were associated with a reduction of the insulin response of 26% for horses on the large pasture and an increase of the insulin response of 5% on the small one (Fig. 2). The regression slopes are compared with individual trends in Fig. 3.

### Correlation between the area under the curve of insulin over time with the serum insulin concentration at 120 min

**Fig. 4:**
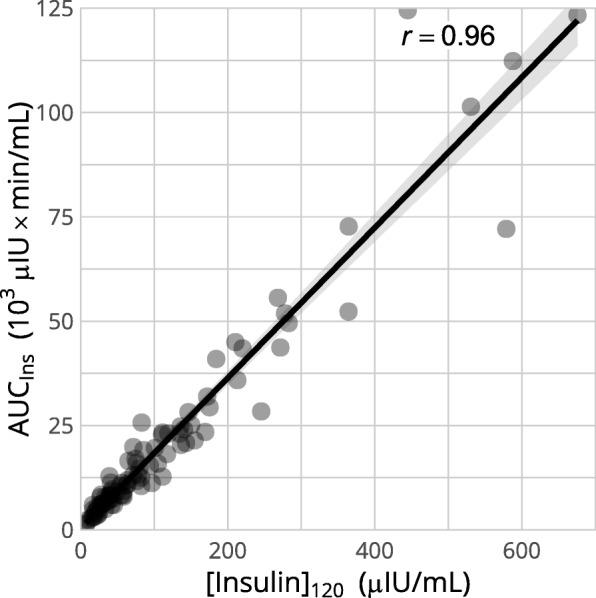
Correlation between the AUC_ins_ and the serum insulin concentration at 120 min ([Insulin]_120_). The correlation between both variables is very high (*r* = 0.96, *p* < 0.001), suggesting that the 120 min value can be used instead of the AUC_ins_ to assess the insulin response to glucose challenges without substantial loss of information

The AUC_ins_ and [Insulin]_120_ expressed a strong linear correlation (*r* = 0.96) (Fig. 4).

## Discussion

The aim of this study was to describe the relationship between weight variations and the insulin response to a glucose challenge. Over a period of nearly 1 year, 19 horses were weighed and subjected to OGTs at regular intervals. Important weight variations – mostly weight loss – were observed. These variations are attributable to unmonitored changes in feed quality and voluntary physical activity. The relative weight significantly predicted the insulin response with 5% weight loss being associated with a reduction of the insulin response of 22%.

When comparing the regression slopes of the relative body weight against the relative insulin response to the trend within individuals (Fig. 3), four horses showing no reduction of the insulin response despite weight loss can easily be identified. These horses came from the smaller pasture, together with horse 3, which was the only stallion. As a conclusion, the causes for the variations in the response to weight loss took place either on the individual level or on pasture level but were compensated by individual factors (like sex) in one horse. By fitting a distinct slope for each pasture, the effect of weight loss became more apparent for horses from the larger pasture, displaying a reduction of the insulin response of 26% for 5% weight loss (Fig. 2).

### Possible causes for the lack of response in some individuals

Neither the evolution of the relative body weight nor of the relative insulin response follow a radically different trend over time between pastures (Fig. 1). When looking at absolute values (data not shown), no statistically significant differences were found between pastures within OGTs. Therefore, the lack of response in most individuals of the smaller pasture appears not to be related to a form of weight loss resistance [13] or a fundamentally higher level of insulin dysregulation, that would be refractory to weight loss. Nevertheless, changing proportions of metabolic active tissues depending on the level of physical activity (e.g. relatively more muscle and less white adipose tissue) could have an influence on the reduction of the insulin response without affecting body weight [23, 24].

Some differences exist in the age and sex distribution between groups. However, the younger group (small pasture) not responding to weight loss appears counterintuitive, since age seems to be positively associated with hyperinsulinemia [25] while sex does not [26]. Yet on the small pasture, the stallion responded to weight loss in contrast to the geldings (Fig. 3), so that there might be an effect of sex on the unknown factor confounded by pasture.

Even though differences in feeding are largely excluded (see Material and methods), there might have been dissimilarities regarding the preferably consumed feed (pasture grasses or hay) or an interaction between body condition and feed intake (both in quantity and quality) [27] that might in turn have affected ID or IR [25, 28–30].

**Fig. 5:**
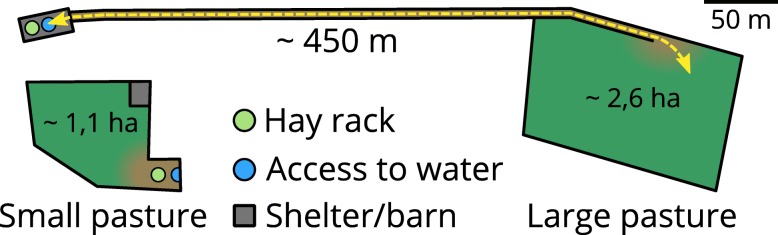
Schematic site plan of the pastures. Five horses were held on a small pasture (on the left) with direct access to shelter, water and hay. Fourteen horses held on a large pasture had to travel approximately 450 m (dotted yellow line) to reach these

Since there were more feeding places than horses on each pasture, an effect of herd hierarchy on available feed appears less likely. Nevertheless, hierarchy could have influenced energy expenditure [27]. As would have the slightly different density of horses on each pasture. Additionally, the layout of the pastures (Fig. 5) differs in such a way that the larger group could have undertaken more voluntary physical exercise than the small one by traveling from the pasture to the shelter several times over the day. The difference in exercising levels would then be responsible for the differences in the response to weight loss. While this corroborates observations from the authors, these differences are not quantifiable due to the retrospective nature of the study. Previous studies have shown, that in the absence of physical activity, an increase of SI through weight loss cannot always be achieved [15]. However, a more stringent diet might help achieve satisfactory results without concurrent exercise [11]. Initial body weight and condition, genotype and the quality of provided forages [13, 31] might explain some differences between these studies. In human medicine, there is also discordant evidence regarding the relative importance of weight loss and exercise in improving SI [32–35].

Lastly, as insulin resistance was not assessed, it cannot be excluded that the horses of either of the groups were less sensitive to insulin, while dysregulated to a similar extent.

### Practical relevance

Standardized testing protocols like the OGT do not induce laminitis. It is therefore difficult to quantify the reduction of insulinaemia required to confer a protection against endocrinopathic laminitis. It has been reported that the [Insulin]_120_ measured during an OGT performed with 0.75 g/kg bwt glucose correlated well with the same measure during grazing, while slightly overestimating the insulin response [36]. The lower glucose dosage (0.5 g/kg bwt) used in this study possibly alleviates this bias, so that a reduction of the insulin response to grazing through weight loss and increased physical activity roughly similar to the reduction observed in the OGT with 0.5 g/kg bwt glucose can reasonably be expected.

In view of the high correlation between [Insulin]_120_ and AUC_ins_ and the linear relationship between rWeight and rAUC_ins_, monitoring the body weight after an initial 0.5 g/kg OGT with only two blood samples (basal and 120 min) could be sufficient to evaluate the evolution of ID. As the implementation of weight loss programs under field conditions might be difficult because of a lack of recognition of obesity [37], concerns regarding welfare under dietary restrictions [5], difficulties in implementing the measures in boarding stables and overall owner compliance [17], the simplified OGT can be repeated to ascertain the effectivity of the measures actually carried out.

### Considerations on the study design and data analysis

Considering the AUC_ins_ an approximation of the level of HI corresponds better to our current understanding of the pathophysiology of ID than the oversimplistic categorisation of healthy and insulin-dysregulated individuals relying on a cut-off. Therefore, no target value to be reached can be given to set up weight loss programs. However safe levels can be determined in weight gain trials [38].

Further limitations are that a rather small and homogeneous population of horses was used and that no quantification of exercise nor diet changes could be performed due to the retrospective nature of the study. As individual variations in the response to weight reduction programs are high [13], it is however not necessarily detrimental to rely on the weight actually lost – which can be measured objectively – rather than estimating the energy expense induced by such programs to predict the reduction in the total insulin response. Lastly, both the magnitude of the correlation between the 120 min insulin concentration and total insulin response, and the nature and strength of the relationship between weight variations and insulin response might differ when other protocols for the OGT than the one described above are used.

## Conclusion

To the authors’ knowledge, this is the first study to demonstrate the linear relationship between the insulin response to a glucose challenge and the weight variations within individuals. These results corroborate the efficacy of weight loss against insulin dysregulation and are relevant for the prevention of laminitis and monitoring of insulin-dysregulated horses in a practical setting.

## Methods

### Horses

Nineteen university owned Icelandic horses of mixed metabolic status were enrolled in this project. For practical reasons five horses (1 stallion, 4 geldings; median age: 17 years, range: 9–17 years) were held as a small herd and fourteen horses (11 mares, 3 geldings; median age: 21 years, range: 14–29 years) were held as a large herd. Over the day, each group had access to neighbouring pastures similar in type and grass abundance (Fig. 5) and were fed hay from the same batches ad libitum. At the end of the experiment, the horses remained in their respective herds.

### Weighing

The horses were weighed 9 to 16 days prior to each OGT with a mobile weighing scale (accuracy: 1%, resolution: 1 kg, precision: 2 kg).

### Oral glucose tests

The OGTs were conducted on site five times at even intervals over a one-year period. The horses were fasted overnight for 8 h before the start of the experiments. In the morning, a jugular vein catheter was aseptically placed for blood collection. After a basal sample had been drawn, 0.5 g/kg bwt glucose were administered via nasogastric tube [39]. Further blood samples were collected 30, 60, 120, 180, and 240 min later. After collection, the samples were immediately transferred into VACUETTE® Serum Clot Activator Tubes.^1^ They were left to clot at room temperature for 20–40 min and then kept at 4 °C. Within 6 h, all samples were centrifuged, aliquoted and stored at − 80 °C until further analysis.

### Insulin measurements

Serum insulin concentrations were measured in duplicates at the end of the experimental phase using an equine-optimized ELISA (Mercodia Equine Insulin ELISA^2^; inter-assay CV 7.7%) previously validated for use in horses [40].

### Statistical analysis

Statistical analysis was performed with R 3.6.1 [41] using the packages afex [42], nlme [43] and emmeans [44]. The area under the insulin curve over time (AUC_ins_) was calculated using the trapezoidal method. The relative weight (rWeight) and the relative AUC_ins_ (rAUC_ins_), both defined as the ratio of the weight or AUC_ins_ of the horse at one OGT to the mean weight or AUC_ins_ of this horse across all OGTs, were used for subsequent analysis to make the results comparable between horses.

A weighted least squares model with individual-level autocorrelation and OGT-level (number of trial) variance structure was fitted to the data with the maximum likelihood procedure using rWeight as single predictor of rAUC_ins_. In a second model, the effect of ‘Pasture’ was added with a Pasture:rWeight interaction. Both models were compared by a likelihood ratio test. Normality of the residuals was ensured visually. Significance level was set at 0.05.

Repeated measures two-way ANOVA was used additionally to describe the evolution of rWeight and rAUC_ins_ over time within and between groups independently. Post-hoc comparisons between groups within time were corrected for multiple comparisons using the Dunnett method.

The correlation between AUC_ins_ and serum insulin concentration at 120 min was assessed using the Pearson correlation coefficient.

## Data Availability

The dataset analysed during the current study is available from the corresponding author on reasonable request.

## Abbreviations

AUC_ins_: Area under the insulin curve over time
CV: Coefficient of variation
EMS: Equine Metabolic Syndrome
ID: Insulin dysregulation
IR: Insulin Resistance
OGT: Oral Glucose Test
rAUC_ins_: Area under the insulin curve relatively to the mean area under the insulin curve of this horse
rWeight: Body weight relatively to the mean body weight of this horse.
SI: Insulin Sensitivity

## Footnotes

1 Greiner Bio-One International GmbH, Maybachstraße 2, D-72636 Frickenhausen, Germany

2 Mercodia AB, Sylveniusgatan 8A, SE-754 50 Uppsala, Sweden
